# Space and time‐resolved monitoring of phosphorus release from a fertilizer pellet and its mobility in soil using microdialysis and X‐ray computed tomography

**DOI:** 10.1002/saj2.20161

**Published:** 2021-01-21

**Authors:** Chiara Petroselli, Katherine A. Williams, Arpan Ghosh, Daniel McKay Fletcher, Siul A. Ruiz, Tiago Gerheim Souza Dias, Callum P. Scotson, Tiina Roose

**Affiliations:** ^1^ School of Engineering, Faculty of Engineering and Physical Sciences Univ. of Southampton Southampton Hampshire SO17 1BJ UK

## Abstract

Phosphorus is an essential nutrient for crops. Precise spatiotemporal application of P fertilizer can improve plant P acquisition and reduce run‐off losses of P. Optimizing application would benefit from understanding the dynamics of P release from a fertilizer pellet into bulk soil, which requires space‐ and time‐resolved measurements of P concentration in soil solutions. In this study, we combined microdialysis and X‐ray computed tomography to investigate P transport in soil. Microdialysis probes enabled repeated solute sampling from one location with minimal physical disturbance, and their small dimensions permitted spatially resolved monitoring. We observed a rapid initial release of P from the source, producing high dissolved P concentrations within the first 24 h, followed by a decrease in dissolved P over time compatible with adsorption onto soil particles. Soils with greater bulk density (i.e., reduced soil porosity) impeded the P pulse movement, which resulted in a less homogeneous distribution of total P in the soil column at the end of the experiment. The model fit to the data showed that the observed phenomena can be explained by diffusion and adsorption. The results showed that compared with conventional measurement techniques (e.g., suction cups), microdialysis measurements present a less invasive alternative. The time‐resolved measurements ultimately highlighted rapid P dynamics that require more attention for improving P use efficiency.

## Core Ideas

- The initial release of P from fertilizer pellets is not well studied
- Microdialysis and X‐ray CT permit noninvasive time‐resolved monitoring
- A lab‐scale setup was used to study solution P concentrations in time and space
- A rapid initial release of P from the pellet was measured, with no replenishment
- P concentration decreased over time, compatible with adsorption onto soil

**Abbreviations**
ICP‐MS: Inductively coupled plasma – mass spectrometry
RR: Relative recovery rates
TSP: Triple superphosphate
XCT: X‐ray computed tomography

## 1 INTRODUCTION

Phosphorus is one of the major limiting nutrients for crop yields (Shen et al., 2011) and global food production relies on fertilizer application (Ashley, Cordell, & Mavinic, 2011). The most widely used source for producing phosphorus fertilizer is rock phosphate (Smil, 2000). However, the global reserve of this is a nonrenewable resource could run out in less than 100 yr (Cordell, Drangert, & White, 2009). Improving fertilizer practices involves maximizing crop production while minimizing costs and avoiding phosphorus losses and subsequent contamination of water bodies (Smil, 2000). In order to optimize these factors, crop plants need to absorb the delivered nutrients efficiently. Plants can only take up P from soil water solutions, but P concentrations in soil solutions are usually very low (<10μM) (Schachtman, Reid, & Ayling, 1998) because of its fast adsorption onto soil particles and microbial degradation processes (Barber, 1995). Precision agriculture can improve fertilization practices by limiting P runoff (Withers, Neal, Jarvie, & Doody, 2014) and minimizing the unintended side effects that contribute to P removal from the soil solution, namely fixation or competition with other plant species (Blackshaw & Molnar, 2017; Grant, Flaten, Tomasiewicz, & Sheppard, 2001). However, in order to develop precision agriculture strategies, it is necessary to gain a fundamental understanding of the movement and behavior of fertilizer products in the soil.

In this context, understanding the spatial and temporal scales of phosphorus mobility in soil is critical for understanding its availability to plants and ultimately for optimizing fertilizer applications. To achieve this, highly resolved spatial measurements of the solute are needed to investigate the localized movement of this low‐mobility nutrient. High temporal resolution is also required for assessing the initial release of P from the inorganic P fertilizer pellet along with the timescales of P transport and immobilization in the soil. However, achieving high spatiotemporal resolution presents some technical challenges. Firstly, low P concentrations in a soil solution (<10μM) (Schachtman et al., 1998) mean that sensitive analytical techniques are required to detect and quantify them. Additionally, to acquire representative and accurate time‐resolved data, the chosen soil solution sampling method should be minimally invasive, allowing the collection of multiple samples without perturbing the experimental setup.

Suction cups are widely used in situ sampling devices for soil solutions. They directly extract the soil solution by inducing lower pressure in the probe than in the soil water, drawing water into the probe through a porous ceramic cup (Göttlein, Hell, & Blasek, 1996). Suction cups come in a range of sizes and they are routinely used for sampling soil solutions both in the laboratory (Gao, Blaser, Schlüter, Shen, & Vetterlein, 2019; Vetterlein & Jahn, 2004) and in field‐scale experiments (Blicher‐Mathiesen, Andersen, & Larsen, 2014). However, as they directly sample the soil solution, they can affect the flow of water through the soil.

Microdialysis provides a minimally invasive and non‐destructive in situ sampling technique designed for sampling small quantities of solutes from living organisms (Bourne, 2003, Hammarlund‐Udenaes, 2017). Microdialysis relies on the principle of passive diffusion of solutes through a semipermeable membrane. Therefore, the device does not actively remove water from the system, reducing its invasiveness compared with suction cups. Although microdialysis is primarily a technique used in pharmacokinetics, and despite the fragility of the probes, it has been successfully applied to soil (Miró & Frenzel, 2004, 2005) and, more recently, to sampling phosphorus from soil solutions (Demand, Schack‐Kirchner, & Lang, 2017; McKay Fletcher et al., 2019a). With microdialysis, the relative recovery rates (RRs) of solutes can be very high, with RRs of >100% previously being reported (Torto et al., 1995), but RRs are highly dependent on the perfusion flow rate, with slower flow rates promoting a higher RR (Inselsbacher, Öhlund, Jämtgård, Huss‐Danell, & Näsholm, 2011; Miró & Frenzel, 2004). The RR also depends strongly on the composition of the perfusion fluid (Buckley, Brackin, Jämtgård, Näsholm, & Schmidt, 2020), the soil moisture (Buckley, Brackin, Näsholm, Schmidt, & Jämtgård, 2017; Jämtgård, Robinson, Moritz, Colgrave, & Schmidt, 2018), and on the physical, chemical, and biological processes that happen in soil (Demand et al., 2017).

**Core Ideas**

- The initial release of P from fertilizer pellets is not well studied
- Microdialysis and X‐ray CT permit noninvasive time‐resolved monitoring
- A lab‐scale setup was used to study solution P concentrations in time and space
- A rapid initial release of P from the pellet was measured, with no replenishment
- P concentration decreased over time, compatible with adsorption onto soil

X‐ray computed tomography (XCT) is a powerful technique for investigating three‐dimensional soil pore space (Lehmann et al., 2006) and in situ root structure (Mooney, Pridmore, Helliwell, & Bennett, 2012). Coupling XCT with nondestructive in situ sampling techniques (e.g., microdialysis) shows promise for elucidating the structural and spatial factors that influence solute transport in porous media, which could ultimately help interpreting the fate of P in the pore space. For example, XCT visualization of microdialysis probes in the soil revealed the presence of a cavity around the membrane caused by the insertion needle used, which could affect the probe's recovery efficiency at different water saturation levels (Brackin, Atkinson, Sturrock, & Rasmussen, 2017). The same study combined microdialysis and XCT to measure nutrient concentrations at different distances from the roots (Brackin et al., 2017). Recently, Gao et al. (2019) used suction cups and XCT for assessing root architecture responses to different spatial distributions of phosphorus fertilizer in soil. They demonstrated a species‐specific response of maize (*Zea mays* L.) root structure to localized P supply in the first 2.5 cm from the fertilizer pellets.

The aim of this work is to gain an insight into the first steps of phosphorus release from a fertilizer pellet and its diffusion into bulk soil via a coupled microdialysis and XCT approach. As the fertilizer pellet is placed in the soil, many processes take place, as reviewed by Hedley & McLaughlin (2005). The initial wetting of the pellet (Hettiarachchi, Lombi, McLaughlin, Chittleborough, & Self, 2006) is followed by the release of P, producing a highly concentrated and strongly acidic solution in the vicinity of the pellet. The transport of the released P can be slowed down by the movement of water towards the pellet and also by the advection of cations that can determine the precipitation of secondary P compounds. Two different concentration zones were established outside the residual pellet (Benbi & Gilkes, 1987), one P‐saturated zone near the pellet where the soil sorption capacity is exceeded and precipitation is the main P immobilization mechanism, and a wider P‐unsaturated zone where P movement is mainly by diffusion. Phosphorus is not expected to travel far from the source because of its slow diffusivity and high susceptibility to adsorption onto soil particles. Therefore, high spatial resolution is required to capture any movement within the soil solution. Moreover, we hypothesize that diffusion timescales are linked to hydraulic connectivity in the soil and the available surface area of soil for adsorption. These, in turn, depend on soil porosity and soil particle size. To test these hypotheses, samples were collected at a high temporal resolution for two different soil particle size distributions in order to assess the influence of soil porosity on phosphorus movement. Subsequently, a comparison with an active soil water sampling technique (suction cups) was carried out in order to assess the effect of the chosen sampling method on the observed patterns of phosphorus movement, particularly the influence of active water flow caused by the suction cups. Understanding these small‐scale processes can give useful information to help optimize the spatiotemporal scales of phosphorus fertilization in terms of both maximizing plant P uptake and minimizing P runoff.

## 2 MATERIALS AND METHODS

### 2.1 Experimental setup

The experimental setup consisted of a 20 ml syringe barrel tube filled with soil kept at close to 100% water saturation by supplying MilliQ water (18 MΩ) from the bottom of the soil column (see Supplemental Figure S1 and Supplemental Figure S2). The soil was kept at 100% saturation in order to maintain a continuous water domain throughout the soil. This meant that anaerobic conditions (and reducing conditions for iron oxides) were maintained during the experimental period. However, had the soil been partially saturated, there would probably have been considerable spatial variation in water saturation. Since the spatial variation in water saturation could not be continuously assessed in this setup, it would not have been possible to control for the effect of a noncontinuous water domain on the movement of P through the system. In addition, microdialysis probes function on the principle of diffusion across a semipermeable membrane and operate best when the semipermeable tip is fully saturated. This is easier to achieve in a soil system that has 100% water saturation than one that has spatial variation in saturation. Preliminary experiments performed watering from the top of the soil column, indicated that watering from the top accelerates P washout in the first 50 h, but the overall trend is comparable (see Supplemental Figure S3). Although watering from the top would give a more realistic representation of what happens in the field, the proposed setup aimed to being as simplified and controlled as possible in order to actively change only one parameter at a time.

The chosen soil was a sandy clay loam Eutric Cambisol, collected from the Ah horizon of an agricultural grassland in the Henfaes Research Station in Abergwyngregyn, Wales, UK (53°14′N, 4°01′W). A summary of the soil properties is presented in Supplemental Table S1. A more detailed description of the soil properties can be found in McKay Fletcher, Keyes, Daly, Van Veelen, and Roose (2019b) and in Oburger, Jones, and Wenzel (2011). Two soil fractions were used in order to investigate the effect of the particle size on P movement. Both soil fractions were obtained by starting from the same soil sieved below 2 mm. The fine fraction comprised all the particles with a diameter below 2 mm; for obtaining the coarse fraction, the particles with a diameter below 1.18 mm were removed. Dry bulk density was $\rho_{b,c}\;$= 1.02 ± 0.01 g ml^−1^ in coarse soil and $\rho_{b,f}\;$= 1.26 ± 0.01 g ml^−1^ in fine soil. The initial soil blank P concentration was 597 ± 16 μg P g^–1^ soil for the coarse fraction and 682 ± 23 μg P g^–1^ soil for the fine fraction. A comparison between the two soil fractions compositions is shown in Supplemental Figure S4. One triple superphosphate (TSP) fertilizer pellet per tube (mass range: 50–60 mg) was used as a localized phosphorus source and positioned 1.5 cm below the soil surface. The average P concentration in the fertilizer used was 2.1 × 10^5^ μg P g^–1^ soil. P was therefore added at a rate of 333 kg ha^−1^.

In situ sampling was performed with microdialysis CMA 11 metal‐free (4 mm) probes (CMA Microdialysis AB, Kista, Sweden) with a 6‐kDa molecular weight cutoff. Microdialysis probes were perfused with MilliQ water (18 MΩ) at a 3.3 μl min^−1^ flow rate (McKay Fletcher et al., 2019a) by a PHD 2000 Programmable Syringe Pump (Harvard Apparatus, Cambridge, UK). Three probes per tube were set up for assessing the spatial scales of phosphorus movement, one placed 0.5 cm above the pellet and the other two placed below the pellet at 1.5 cm and 3.0 cm distance from the P source (Supplemental Figure S2). In the first 12 h of measurements, sampling was performed at a high temporal resolution by perfusing the probes for consecutive 2 h periods (sample volume: 0.396 ml). For the remainder of the 2‐wk experiment, one sample was collected every 24 h by perfusing the probes over a 12‐h period (sample volume: 2.376 ml).

MicroRhizon suction cup samplers, whose ceramic tips had a mean pore size of 0.15 μm (Rhizosphere Research Products B.V., Wageningen, The Netherlands), were compared with microdialysis probes. Suction cups were deployed in replicate setups at the same positions as the microdialysis probes and operated at a 33 μl min^–1^ flow rate by the same syringe pump. A fixed volume of 0.6 ml of soil solution for each probe was sampled every 24 h. High‐resolution sampling was not performed because of the volume of water removed from the soil column at each timepoint in relation to the total volume of water present in the system. Both the microdialysis (dialysates) and suction cup samples were kept sealed and refrigerated (+4 °C) until analysis.

In order to assess the baseline P values for the chosen soil, a blank soil treatment was set up with the same characteristics and sampling procedure as the others but without the pellet. Phosphorus concentrations in dialysates from the blank soil were consistently lower than the detection limits (data not shown); in the suction cup samples, the blanks were two orders of magnitude higher than the limit of detection but were still two orders of magnitude lower than the fertilizer treatment data.

The two sampling techniques were intercalibrated in order to obtain comparable values. Calibration standards were made by extraction of ground TSP pellets in MilliQ water, filtration of the obtained solution, and subsequent dilution of the stock concentration. A four‐standard calibration was performed for both the microdialysis and suction cup samples; the calibration curves are shown in Supplemental Figure S5. The data show that absolute recovery via microdialysis decreases with increasing phosphorus concentrations (Figure S5). A correction factor derived from calibration experiments was therefore applied to all the microdialysis data in order to normalize them to effective soil solution concentrations. Furthermore, probe intervariability was evaluated by replicating the calibration with three different probes of each kind. Microdialysis probes show higher variability (with a SD of 6% the mean) than suction cups (2%).

### 2.2 Quantitative phosphorus determination by inductively coupled plasma – mass spectrometry

Inductively coupled plasma – mass spectrometry (ICP‐MS) analyses were performed at the School of Ocean and Earth Science, University of Southampton in a quadrupole ICP‐MS (XSeries2, Thermo‐Scientific, ThermoFisher‐Scientific, Oxford, UK). Thirteen elements were quantified, namely Na, Mg, Al, Si, P, K, Ca, Mn, Fe, Cu, Zn, Sr, and Pb. Calibration was performed with seven custom synthetic standard solutions prepared from single‐element standards (Inorganic Ventures, Christiansburg, VA, USA). All samples and standards were prepared in 3% sub‐boiled nitric acid spiked with internal standard elements (Be, In, and Re) to correct for drift and matrix effects during the ICP‐MS measurements. The dialysate and suction cups samples were diluted by 25‐fold prior to analysis to fit within the measurement range of the instrument.

At the end of the experiment, the total P concentration at different depths along the column (both in solution and bound to the soil) was measured in the following way. The soil columns were isolated from the watering system by closing the tap and immediately frozen, standing vertically, without allowing water to drain or dry. The frozen soil columns were sliced at a 0.5‐cm resolution with a ceramic knife, then the obtained samples were air‐dried for 48 h and oven‐dried at 65 °C overnight. An aliquot of approximately 100 mg of the oven‐dried sample was weighed for digestion. The soil digestions were performed via a combined aqua regia and HF–HClO_4_ protocol to give total digestion (see Supplemental Material). The final solutions were subsampled, dried and dissolved for analysis.

### 2.3 X‐ray computed tomography

X‐ray computed tomography scans were carried out to visualize changes in pellet structure over time and to demonstrate that all the components in the microdialysis setup could be visualized in three dimensions by XCT. Scans were carried out with a modified 225 kVp Nikon/Xtek HMX CT scanner (Nikon Metrology UK Ltd., Nottingham, UK) at the Mu‐VIS imaging centre, University of Southampton, UK. Each scan used 1,601 projections, with eight frames per projection and a 134‐ms exposure per frame. The energy chosen was 120 kV at 44 W. The resulting voxel size was 20 μm. These parameters were chosen to provide sufficient contrast to visualize the differences among the soil, soil water, probes, and fertilizer pellet while minimizing the scan time (each scan took approximately 30 min). The voxel size was the smallest possible so that the entire column fitted within the field of view, to give the highest resolution possible. Scans were reconstructed with a filtered back‐projection algorithm in CTPro (Nikon). Images were processed using ImageJ, a collaborative, open‐source platform for image analysis, developed in Rueden et al. (2017) and Schneider, Rasband, and Eliceiri (2012b), and distributed as FIJI, which includes a range of useful plugins (Schindelin et al., 2012a).

### 2.4 Model parameter estimation

The parameter estimation for diffusion of P through soil and its adsorption onto soil particles is based on the model presented by McKay Fletcher et al. (2019a). This model describes the evolution of P concentration in soil solution (dissolved P) as well as the concentration of P adsorbed onto the soil particles. However, unlike McKay Fletcher et al. (2019a), we do not consider the removal of P by the microdialysis probes. At any time *t* and depth *z* in the sample, we denote the concentration of dissolved P as $c_{sol}\left(z,t\right)$ and adsorbed P as $c_{ad}\left(z,t\right)$. The model that we use is given by the following equations:

(1) $$\begin{array}{ccc} \frac{\partial c_{\mathrm{sol}}}{\partial t}\left(z,t\right) & = & D\frac{\partial^{2}c_{\mathrm{sol}}}{\partial z^{2}}\left(z,t\right)-\beta_{1}c_{\mathrm{sol}}\left(z,t\right)\\ & & +\;\beta_{2}c_{\mathrm{sol}}\left(z,t\right),0<z<L,0<t<T \end{array}$$

(2) $$\frac{\partial c_{\mathrm{ad}}}{\partial t}\left(z,t\right)=\beta_{1}c_{\mathrm{sol}}\left(z,t\right)-\beta_{2}c_{\mathrm{ad}}\left(z,t\right),0<z<L,0<t<T,$$

where *D* (m^2^ s^−1^) is the effective diffusivity of P in the sample (including geometric impedance but not the buffer power of soil), β_1_ (s^−1^) is the rate of adsorption of dissolved P onto the soil particles, β_2_ (s^−1^) is the rate of desorption of P from the soil particle surfaces to the solution, *L* is the total depth of the column used in the setup, and *T* is the total duration of the experiment. The model assumes that no amount of P is lost or gained in the system. This implies that any loss of P resulting from sampling is considered negligible. It is also assumed that P disperses through the soil by diffusion only.

The boundary conditions for Equation (1) are taken to be no flux of P through either the top or bottom surfaces, in accordance with the experimental setup, as expressed by Equation (3) and Equation (4), below, which indicate time intervals when the boundary conditions:

(3) $$\frac{\partial }{\partial z}c_{\mathrm{sol}}\left(0,t\right)=0,0<t<T$$

(4) $$\frac{\partial }{\partial z}c_{\mathrm{sol}}\left(L,t\right)=0,0<t<T.$$

Given the above boundary conditions, the one‐dimensional model equations can be uniquely solved for any given initial concentrations $c_{sol}\left(z,0\right)$ and $c_{ad}\left(z,0\right).$

We numerically estimated the model parametersD, β_1_, and β_2_ for coarse soil by using data from the experiments via the following methodology. Let us denote the depths of the three probes as *l*_1_, *l*_2_, and$\;l_{3}$. Let us also denote the mean value of the data at sampling time $t_{j}$ for the probe at depth $l_{i}$ as $\phi_{ij}$ and the corresponding standard deviation as$\;\sigma_{ij}$.

The parameters in Equations (1) and (2) are estimated by minimizing the error functional *R* described by Equation (5):

(5) $$R=\sum_{i,j}\frac{{\left\|c_{\mathrm{sol}}\left(l_{i},t_{j}\right)-\phi_{ij}\right\|}^{2}}{\sigma_{ij}^{2}}$$

The minimization was performed by the MATLAB function “fmincon”; the partial differential equations for $c_{sol}$ and $c_{ad}$ were solved at each optimization step via a finite difference approximation and the MATLAB function “ode45”.

## 3 RESULTS AND DISCUSSION

### 3.1 Phosphorus pellet dissolution

**FIGURE 1:**
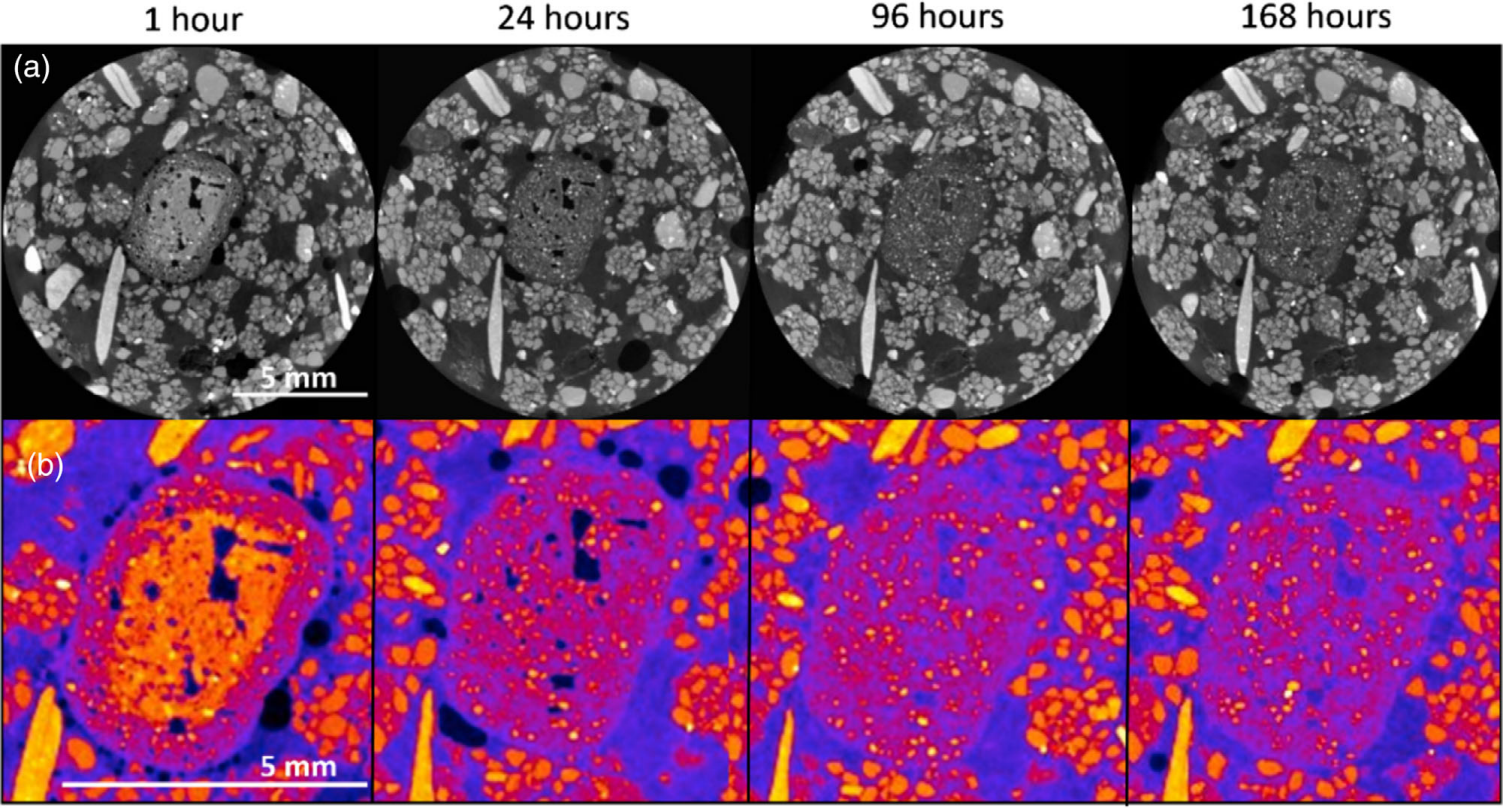
Triple superphosphate (TSP) pellet dissolution in coarse soil visualized by X‐ray computed tomography imaging. The top row is reconstructed gray value slices through the same region of the pellet at different time points; the lower row is the pellet enlarged and a heatmap lookup table (Fire in ImageJ) applied to the pellet to accentuate the differences in gray values

The XCT data (Figure 1) demonstrate that the TSP pellet remained in place for the 8 d that the soil was scanned for. However, the appearance of the pellet changed over time, particularly in the first 24 h. One hour after wetting, the pellet had two distinct bands of X‐ray attenuation, showing water infiltrating partway into the pellet. After 24 h, the solid parts of the pellet were completely wetted, though pockets of air remained. The initial pellet wetting was rapid and occurred within the first 24 h, maximizing the diffusion pathway for P throughout the pellet to the soil. The pellet reduced in X‐ray attenuation by 96 h and had become increasingly difficult to distinguish from the surrounding soil. We interpreted this as a sign of dissolution, although the pellet changed very little in geometry. Therefore, either no dissolution occurred within the experimental period or else, although P dissolved and diffused away from the pellet, parts of the pellet were insoluble and the pellet therefore remained geometrically sound. This second hypothesis is supported by the observations by Hettiarachchi et al. (2006), who observed, via XCT, a decrease in pellet density, along with an increase in its porosity over time, and attributed that to the progress of water absorption by the pellet and dissolution of the inorganic P compound. No further changes were recorded after 96 h, with the pellet showing the same attenuation after 168 h.

### 3.2 Phosphorus space‐ and time‐resolved patterns

**FIGURE 2:**
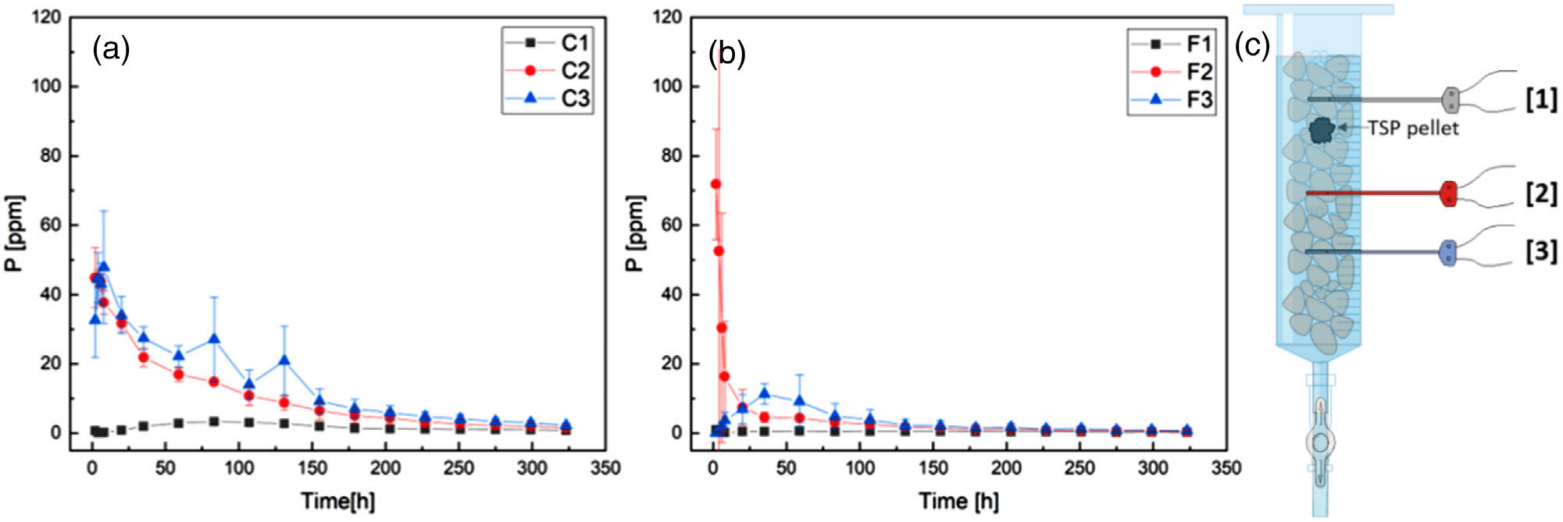
Microdialysis results for (a) coarse soil (1.18–2 mm in diameter) and (b) fine soil (<2 mm in diameter). Averages and SDs are shown for each probe. (c) Schematic of the setup. Probe 1 (gray) is positioned 0.5 cm above the pellet; Probe 2 (red) and Probe 3 (blue) are positioned below the pellet at 1.5 and 3 cm distance from it, respectively

Figure 2a shows the time‐resolved data from the three microdialysis probes at different depths in the coarse soil experiment. The two probes positioned below the pellet (C2 and C3) showed the same temporal pattern and comparable P concentrations: an initially very high concentration of P, which subsequently decayed exponentially. This pattern suggests an “instantaneous” (below sampling frequency) release of P from the pellet that occurred within the first 2 h of wetting the soil, followed by a P concentration decrease in soil solution over time, which can be attributed to different processes that determine the immobilization of P, like its adsorption onto soil particles. This “instantaneous” release is supported by the rapid wetting of the TSP pellet shown in the XCT scans (Figure 1). Probe C1, which was positioned above the fertilizer pellet, showed a slower increase in P concentration in the soil solution (over the first 83 hours), followed by a decrease compatible with adsorption onto soil particles. Moreover, the maximum P concentration detected by the probe positioned above the pellet (C1; 3.3 ± 0.7 μg P ml^–1^ dialysate solution) was one order of magnitude lower than the concentrations detected by the probes below the pellet (C2 and C3; 45 ± 8 and 48 ± 16 μg P ml^–1^ dialysate solution), suggesting that P movement is favored in the downward direction, probably because of gravity. This hypothesis was tested with a set of experiments in nonstirred water columns, which showed that P concentrations increased 10 times faster in the downward than in the upward direction. Details and data from this experiment can be found in Supplemental Figure S6.

The effect of soil particle size on the phosphorus dispersion rates was investigated. Estimating the mean particle density with the density of quartz ($\rho_{s}\;$= 2.65 g ml_solid_^−1^), we estimated the soil porosity as $\phi \;=\;1-\frac{\rho_{b}}{\rho_{s}}$; thus an overall decrease in soil porosity from $\phi_{c}=\;0.614$ L_pore_ L_bulk_^−1^ to $\phi_{f}=\;0.525$ L_pore_ L_bulk_^−1^ was observed. This change in soil porosity leads to a decrease in the effective diffusion of P in soil resulting from decreased soil pore volume (Barraclough & Tinker, 1981). However, the increase in soil surface area could play a role as well, because of P adsorption onto soil particles. We therefore expect an overall nonlinear decrease in the effective diffusion of P in the fine soil case because of the sum of these two processes.

The results from the experiment conducted in fine soil (particle diameter below 2 mm) are shown in Figure 2b. The trend detected by the probes below the pellet (F2 and F3) confirms the “instantaneous” release of a pulse of P from the pellet. Probe F3, which was positioned 3 cm below the pellet, captured the whole profile of the pulse, the start of which was missed in coarse soil and in probe F2, resulting in a high apparent initial P concentration. In the fine soil, this pulse lasted 125 h. The time shift between the maximum concentration recorded by Probe F2 and Probe F3 is about 31 h and the maximum P concentrations detected by Probe F3 (11 ± 3 μg P ml^–1^ dialysate solution) are almost one order of magnitude lower than those detected by Probe F2 (72 ± 16 μg P ml^–1^ dialysate solution). These results support the hypothesized role of smaller particles in slowing down the movement of phosphorus through the soil column. In fact, more effective packing of small particles can lead to a decrease in soil porosity and connectivity, together with the increased tortuosity of the diffusive paths, thus slowing down the movement of solutes through soil. Moreover, colloidal particles can adsorb high quantities of P because of their high surface area to mass ratio and can contribute to its mobility by particle diffusion through soil (de Jonge, Kjaergaard, & Moldrup, 2004). All of these processes affect the speed of P movement in the soil, they can affect the adsorption–desorption equilibrium, determining a different distribution of P associated with soil particles in the soil column. Above the fertilizer pellet, the P concentration values were still above the limit of detection; however, no clear concentration trend was recorded. This was caused by higher variability among the three replicates, attributed to the lower soil homogeneity indicated by the presence of different sized particles in the system, and to lower control of the reproducibility of the soil column packing stage.

### 3.3 Total P concentration profile

**FIGURE 3:**
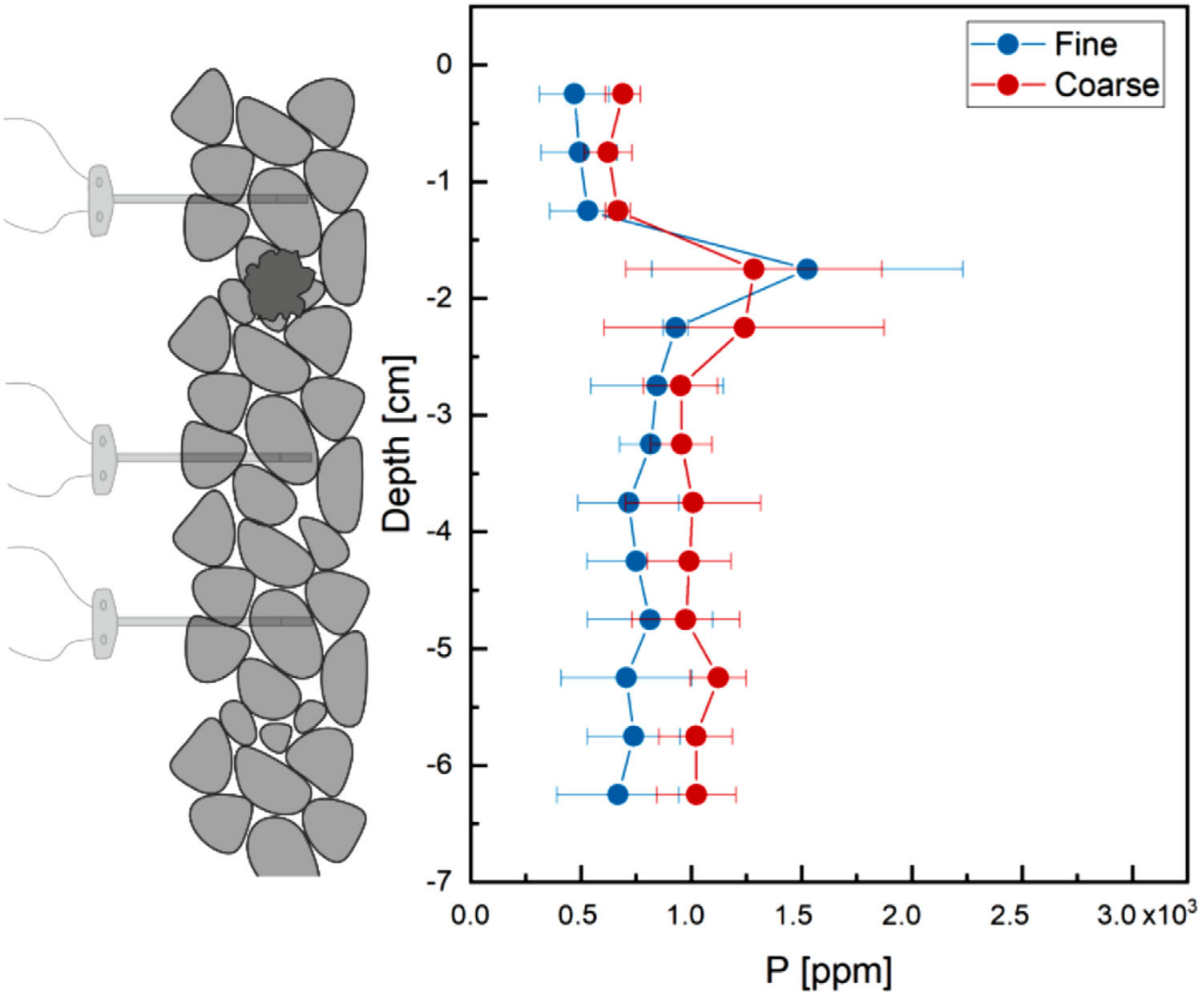
Total P concentration profiles in the soil columns at the end of the experiment, measured by soil digestion. Fine soil is sieved below 2 mm; coarse soil between 1.18  and 2 mm. Errors are reported as SDs over three replicates

Total P concentrations in both soil types were recorded at the end of the experiment by total soil digestion, followed by ICP‐MS analysis. Total P concentrations, expressed as μg P g^–1^ soil for each layer in coarse and fine soil, are shown in Figure 3. The TSP pellet was located 1.5 to 2 cm from the top of the soil column and its presence was indicated by the spike in P concentration in both soil types. Phosphorus concentrations above the pellet were consistently lower than those below the pellet in both soil types, confirming the trend observed in the soil solution and demonstrating the overall effect of gravity on P distribution. In coarse soil, P movement was promoted in both the upward and downward directions from the pellet, producing higher concentrations in all the layers and lower concentration in the pellet layer than in fine soil. On the other hand, fine soil impedes the movement, causing more P to be trapped in the pellet layer and determining decreasing concentrations at increasing distances from the pellet.

Overall, the experimental data shows a faster diffusion of P from the fertilizer pellet to bulk soil, faster pulse movement, and a more homogeneous distribution of P over the soil column in the coarse case than in the fine one. This experiment showed that among all the complex processes regulating P mobility in soil, a finer soil particle size distribution contributes to actively slow down the pulse; a decreasing P concentration trend in soil at the end of the experiment was also observed with increasing distance from the P source.

### 3.4 Modeling P movement patterns in coarse soil

Data for the coarse soil (Figure 2a) showed high initial concentrations (i.e. after the first 2 h) for both the probes positioned below the pellet. After the first 8 h, a steady diffusion pattern could be observed for these two probes. This suggests that the concentration pulse emanated by the fertilizer pellet dispersed down the column and settled into a certain distribution early in the experiment. We estimated the parameters *D*, β_1_, and β_2_ by fitting the data recorded after 8 h from the start of the experiment. We used an effective continuous initial distribution that would best explain the eventual distributions observed in the data. Running iterations of the model and using values close to the estimated values of the parameters given in McKay Fletcher et al. (2019a), we noticed that the initial profile of P concentration in soil solution was reflected in the final profile of total P concentration, which is mainly determined by P adsorbed onto soil particles, suggesting that adsorption occurs at a higher rate than diffusion. Based on the soil digestion data (total P concentrations), we assumed a Gaussian profile for the initial concentration of dissolved P, with its peak value *c*_pulse_ at the initial position of the pellet, and variances of *a* ^2^ above the pellet and *b* ^2^ below the pellet to a depth of 27.5 mm. Below this, we assumed a constant uniform profile for the initial concentration of dissolved P through to the bottom. The parameters *c*_pulse_, *a*, and *b* were also determined by optimization of the function R, as defined in Equation (5). The initial distribution of the concentration of adsorbed P [i.e., c_ad_ (*z*,0)] was taken to be a uniform prefertilization equilibrium value. The expressions for *c*_sol_ (*z*,0) and *c*_ad_ (*z*,0) are provided in Equations S1 and S2 in the Supplemental Material. The initial distribution profile of dissolved P is shown in Supplemental Figure S7, where the estimated values of *c*_pulse_, *a*, and *b* have been used.

Table 1 shows the estimated values of the model parameters, along with the corresponding initial values used to initialize the optimization algorithm. With the estimated values of the model parameters, the model fits the data with 22% error.

**TABLE 1:** Initial values of the optimization parameters

Parameter	Description	Initial guess	Estimated value	Units
*D*	Diffusion rate	$1.9\;\times 10^{-11}$	$1.6\;\times 10^{-11}$	$m^{2}\;s^{-1}$
β_1_	Adsorption rate	$3.1\;\times 10^{-6}$	$2.7\;\times 10^{-6}$	s^−1^
β_2_	Desorption rate	$3.6\;\times 10^{-9}$	$5.2\;\times 10^{-9}$	s^−1^
$c_{pulse}$	Maximum strength of the initial pulse	$8.5\;\times 10^{2}$	$9.0\;\times 10^{2}$	μg P ml^–1^
*a*	Spread of initial pulse above the pellet	3.0	3.0	mm
*b*	Spread of initial pulse below the pellet	12	12	mm

**FIGURE 4:**
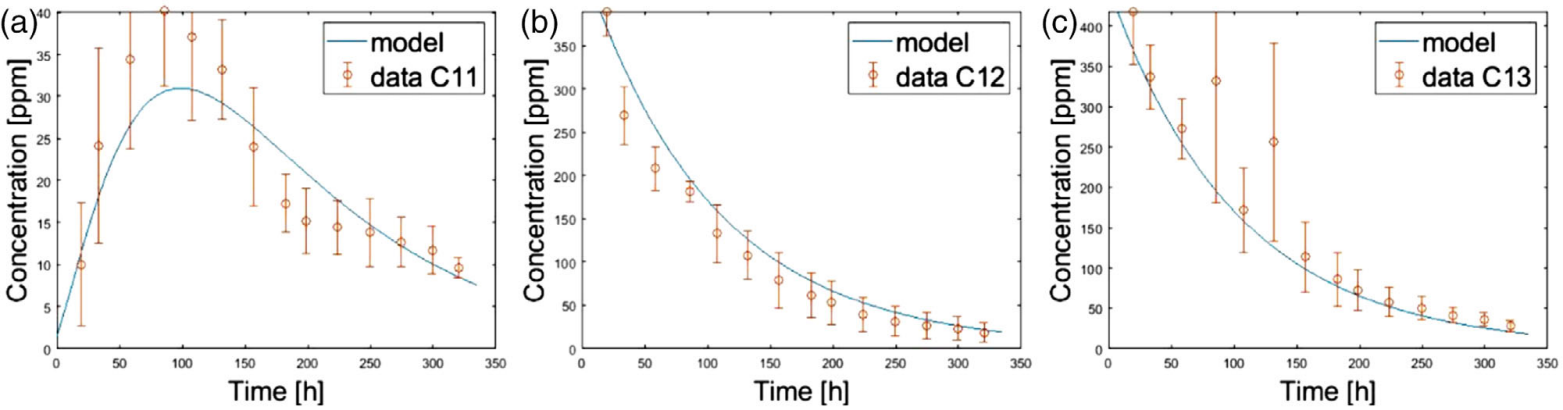
Microdialysis data in coarse soil with the SD in red and the fitted curve in blue. Panel (a) corresponds to Probe 1 positioned 0.5 cm above the pellet, Panel (b) corresponds to Probe 2 positioned 1.5 cm below the pellet, and Panel (c) to Probe 3 positioned 3.0 cm below the pellet

Figure 4 shows the model fit of the P concentrations recorded by the three microdialysis probes in coarse soil. As can be observed in Figure 4, the concentrations of dissolved P predicted by the model with the estimated parameters are close to the recorded data and mostly lie within the standard deviations of the data points. At the top probe position, the model predicted an initial rise in dissolved P concentration resulting from diffusion of the pulse, then a subsequent decrease in concentration caused by adsorption of P onto the soil, all in agreement with the data. At the bottom two probe positions, the model predicts adsorption to be the dominant process, resulting in an exponential decrease in the dissolved P concentrations, once again in close agreement with the observed data.

### 3.5 Comparison between microdialysis probes and suction cups

**FIGURE 5:**
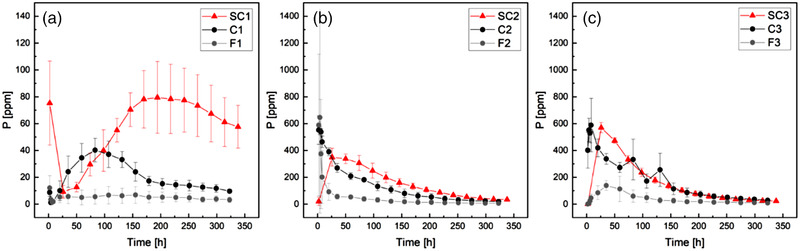
Comparison between suction cups (red, fine soil) and microdialysis in fine (gray) and coarse (black) soil. Averages and SDs are shown for each data point. The left‐hand panel shows Probe 1 positioned 0.5 cm above the pellet, the central panel shows Probe 2 positioned 1.5 cm below the pellet, and the right‐hand panel shows Probe 3 positioned 3 cm below the pellet

The effect on P movement of passive sampling from microdialysis was compared with the active uptake of the soil solution by suction cups in an experiment on fine soil. The phosphorus concentrations and the trend detected from the two suction cups positioned below the pellet were compatible with the microdialysis results (Figure 5b,c). However, although suction cup sampling was carried out in fine soil, the recorded concentrations more resembled the ones detected by microdialysis in coarse soil. This suggests that advection caused by the active suction of the devices transports P through the fine soil at rates comparable with the passive transport monitored via microdialysis in the coarse soil. In addition, above the pellet (Figure 5a), microdialysis in fine soil recorded no trend in P concentration. Suction cups, on the other hand, recorded a clear trend with a delayed peak with respect to the one detected by microdialysis in coarse soil: P concentrations rose slowly over the first 175 h, reached a peak, and started declining again. The discrepancy between microdialysis and the suction cups suggest that water movement caused by the active sampling technique could lead to an overestimation of P mobility. It is worth noting that the temporal resolution of suction cups in the first 12 h is lower than that of the microdialysis experiment because of the active uptake of soil water by the suction cups. Because of this resolution limitation, the maximum concentration recorded 1.5 cm below the pellet (SC2) was lower than at 3 cm below the pellet (SC3) at the same timepoint, despite being closer to the pellet. Moreover, it was not possible to capture the entire pulse profile, as was achieved with microdialysis. Therefore, it was not possible to quantify the effect of the active sampling procedure on counteracting the decreased movement of the pulse caused by fine soil particles and that it is possible to overestimate P mobility in fine soil. A thorough modeling approach that accounts for the pellet dissolution rate (Ruiz et al., 2020) and for probe uptake in the different sampling techniques could shed some light on these aspects, as could further studies dealing with device specific‐sampling methodology.

The suction cup experiment allowed our microdialysis results to be compared with a similar study that used suction cups to investigate plant responses to localized versus homogeneous P fertilizer distribution (Gao et al., 2019). The authors observed very high P concentrations in the localized P treatment on the first day, followed by a decrease in concentration over time. Despite our use of microdialysis, the different scales of the setup, and the plant in their system, our results are all compatible with what they observed below the pellet. Gao et al. (2019) also observed a maize root response to the localized phosphorus supply in the first 2.4 cm below the fertilizer pellets layer, though no increased root biomass was observed above the layer. We hypothesize that the preferential movement of P in the downward direction observed in this study induces root proliferation in the area below the pellet but the concentrations above the pellet do not reach the required levels to induce a plastic response (Drew, 1975).

In summary, we developed an assay for monitoring phosphorus release from a fertilizer pellet and its movement into bulk soil. Microdialysis allows for space‐ and time‐resolved measurements of P in soil solutions and yields result comparable with the popular suction cups, which are more invasive. The results show a rapid release of P from the fertilizer pellet, which happens with a narrower timescale than the sampling resolution, followed by a decrease over time compatible with adsorption onto soil particles. For agricultural applications, this might indicate that there are only narrow time windows after fertilizing when actions can be taken to mitigate P pollution. This is also a point that requires more research and further verification at larger spatial scales, as it is not clear to what extent P is able to transport through the soil domain. Furthermore, modeling of P diffusion and adsorption onto soil particles reproduced the experimental data and allowed us to determine P diffusion coefficients in the soil. Future studies could enhance these models to consider different convective fluxes associated with changes in soil moisture, water uptake by suction cups, and the influence of gravitation on P transport in the soil domain.

## AUTHOR CONTRIBUTIONS

CP and KAW contributed equally to this study. CP conducted the experiments, carried out the analytical lab work, analyzed the data, participated in designing the study, and drafted the manuscript. KAW participated in designing the study, conducted the computed tomography scans, analyzed the data, participated in the lab work, and critically revised the manuscript. AG conducted the modeling work and helped draft the manuscript. DMF, SAR, and TGSD participated in the modeling work and critically revised the manuscript. CPS participated in designing the study and helped with the lab work. TR conceived the study, coordinated the study, and critically revised the manuscript.

## CONFLICTS OF INTEREST

The authors declare that there is no conflicts of interest.

## Supporting information

Supplemental Figure S1. System for maintaining water saturation
Supplemental Figure S2. Experimental setup scheme
Supplemental Figure S3. Phosophrus concentrations in the three probes from the tube watered from the top (closed symbols) and from the bottom (open symbols)
Supplemental Table S1. General properties of the Eutric Cambisol soil
Supplemental Figure S4. Concentration of the studied elements in blank soil
Supplemental Figure S5. Calibration curves for microdialysis (red) and suction cups (blue) compared with calibration standard concentrations (gray).
Supplemental Figure S6. Phosphorus concentration trend in nonstirred water columns.
Supplemental Figure S7. Initial distribution of dissolved P concentration along the soil column

## Data Availability

Data from this study are available at https://doi.org/10.5258/SOTON/D1377.
