# Supplementary material for: Space and time‐resolved monitoring of phosphorus release from a fertilizer pellet and its mobility in soil using microdialysis and X‐ray computed tomography
Source: Soil Sci Soc Am J. 2021 Jan 21;85(1):172–83. doi: 10.1002/saj2.20161 (PMC8611795; doi:10.1002/saj2.20161)
Supplement: Supplementary file 1 — Supplemental Figure S1. System for maintaining water saturation Supplemental Figure S2. Experimental setup scheme Supplemental Figure S3. Phosophrus concentrations in the three probes from the tube watered from the top (closed symbols) and from the bottom (open symbols) Supplemental Table S1. General properties of the Eutric Cambisol soil Supplemental Figure S4. Concentration of the studied elements in blank soil Supplemental Figure S5. Calibration curves for microdialysis (red) and suction cups (blue) compared with calibration standard concentrations (gray). Supplemental Figure S6. Phosphorus concentration trend in nonstirred water columns. Supplemental Figure S7. Initial distribution of dissolved P concentration along the soil column [file SAJ2-85-172-s001.docx]

**Supporting Information for *“Space and Time-Resolved Monitoring of Phosphorus Release from a Fertilizer Pellet and its Mobility in Soil Using Microdialysis and X-ray Computed Tomography“***

Chiara Petroselli*^1^, Katherine A. Williams*^1^, Arpan Ghosh^1^, Daniel McKay Fletcher^1^, Siul A. Ruiz^1^, Tiago Gerheim Souza Dias^1^, Callum P. Scotson^1^, Tiina Roose^1^

^1^University of Southampton, Faculty of Engineering and Physical Sciences, Southampton (UK)

1. Experimental setup


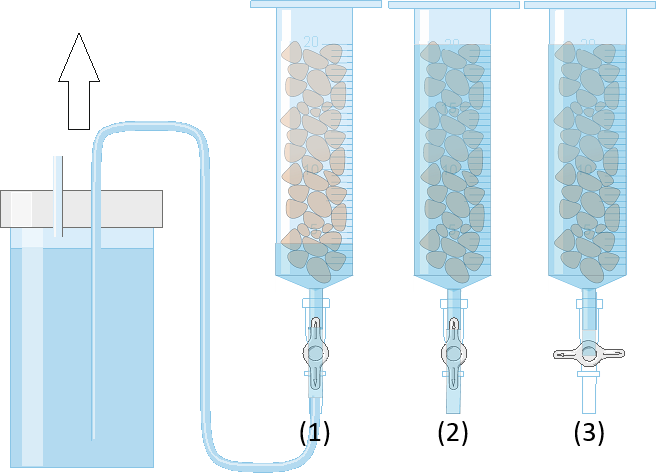


Figure S1 – System for maintaining water saturation. (1) A reservoir of water was connected to the base of the tube and lifted to raise the water level to the soil surface. (2) the water level was maintained at the soil surface. (3) The stopcock can be closed to prevent water from leaking during CT scanning.


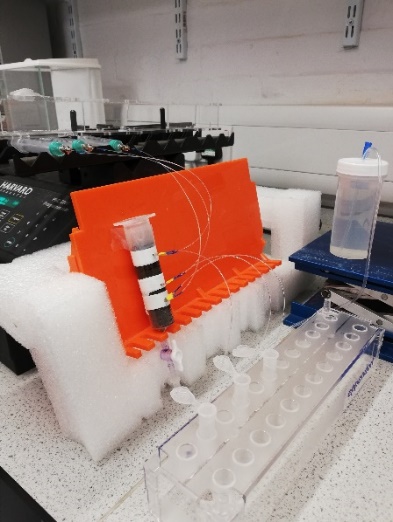


**(a)**

**(b)**


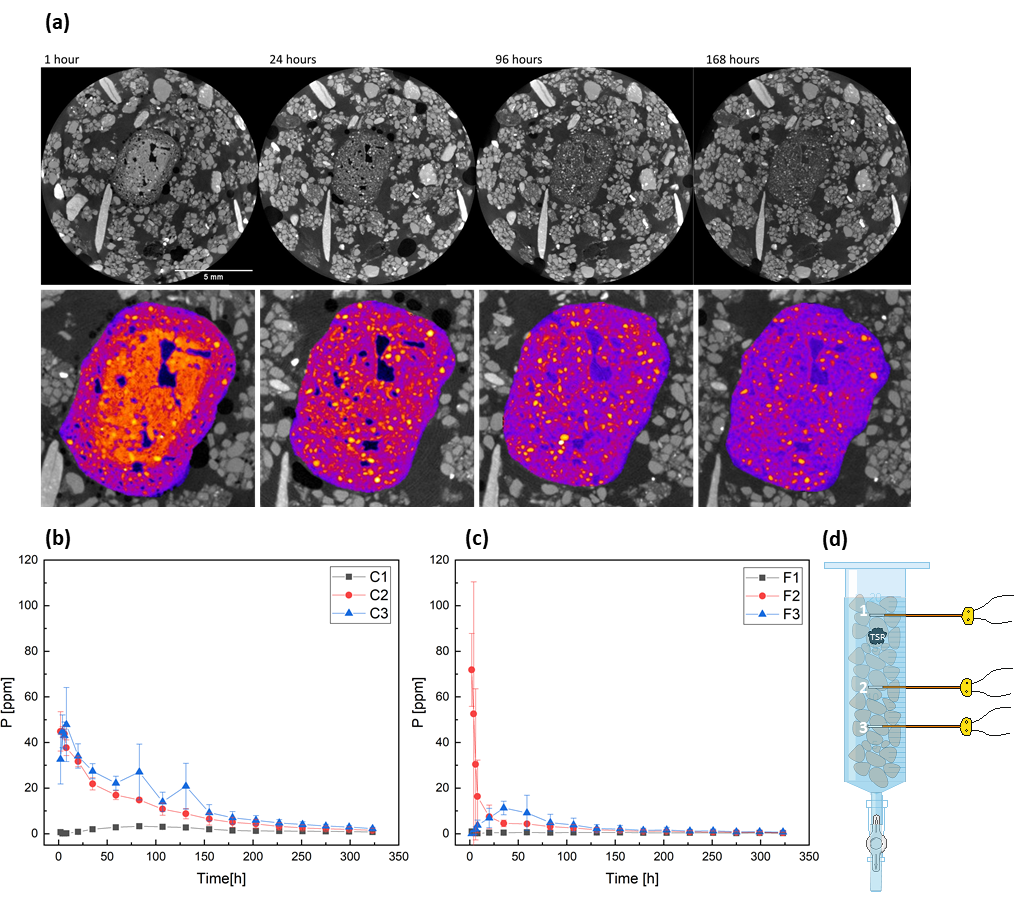


Figure S2 – Experimental setup scheme (a). Micro samplers are positioned 5 mm above and 15 and 30 mm below the TSP fertilizer pellet, respectively. Panel (b) shows a picture of the working setup.

2. Effect of watering direction on P movement

Gao et al. 2019 observed a decreasing trend in phosphorus concentrations on samples collected via suction cups. They hypothesized a role of the water addition from the top of the soil column on the phosphorus trend, with the water flow washing the P released from the pellet in the downwards direction. Since our first set of experiments was done by watering from the top of the soil column and the same trend was observed, we decided to test this hypothesis by setting up a system where the water saturation was achieved and maintained by watering from the bottom of the soil column. Results from the two setups are compared in Figure S3, showing comparable concentrations. However, a steeper P decrease is observed in the watering from the top case in the first 50 h that eventually evens out after 75 h. We therefore conclude that the overall trend observed in the two cases is comparable, and that watering from the bottom allows to avoid accelerated P washout towards the bottom of the soil column. As watering from the bottom also allows for a more controlled water saturation level throughout the whole soil column, it was chosen for the subsequent experiments.


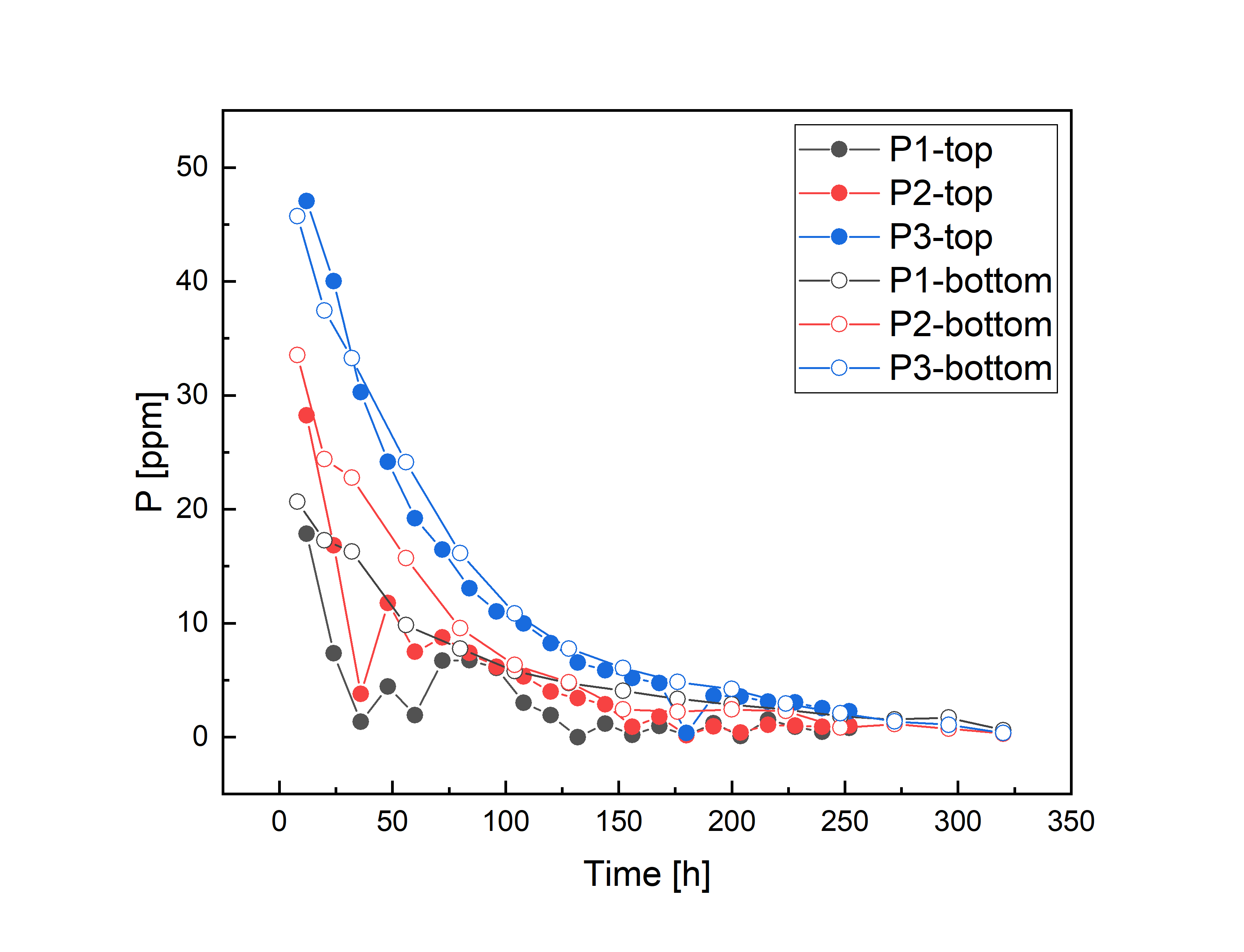


Figure S3 – P concentrations in the three probes from the tube watered from the top (closed symbols) and from the bottom (open symbols). Probes 1, 2 and 3 are positioned 0.5 cm, 1 cm and 2.5 cm below the P pellet, respectively.

3. General properties of the Eutric Cambisol soil used in the experiments.

Table S1 shows measurements of physical and chemical properties of the Eutric Cambisol collected in the Henfaes Research Station in Abergwyngregyn, Wales. Soil mineralogical composition is expressed as a percentage, while soil nutrients are reported as concentrations on a dry soil mass basis. Mean and Standard Error of the Mean (SEM) are reported. Data of Fe, Al and Ca (total, crystalline and amorphous) are taken from Oburger, Jones et al. (2011). Crystalline concentrations were determined using a citrate-dithionate-bicarbonate (CDB) method (Jackson, Lim et al. 1986), while amorphous concentrations using an acid-ammonium-oxalate extractable (AAO) method (Loeppert and Inskeep 1996). Available phosphorus was extracted using 0.5 M acetic acid in a soil-to-solution ratio (SSR) of 1:10 (w/v) (Oburger, Kirk et al. 2009).

Table S1 – General properties of Eutric Cambisol soil

| Property | Mean ± SEM |
| --- | --- |
| pH_(H2O)_ | 6.12 ± 0.05 |
| Electrical conductivity (µS cm^-1^) | 26.5 ± 0.1 |
| Water holding capacity (g kg^-1^) | 356 ± 6 |
| Total C (g kg^-1^) | 25.35 ± 1.47 |
| Total N (g kg^-1^) | 2.95 ± 0.06 |
| Clay (%) | 20 |
| Silt (%) | 37 |
| Sand (%) | 43 |
| Crystalline Fe/Al (CBD) |  |
| Fe (g kg^-1^) | 1.4 ± 0.1 |
| Al (g kg^-1^) | 1.4 ± 0.1 |
| Amorphous Fe/Al (AAO) |  |
| Fe (g kg^-1^) | 5.0 ± 0.1 |
| Al (g kg^-1^) | 1.6 ± 0.0 |
| Total (Aqua regia) |  |
| Fe (g kg^-1^) | 46 ± 0.5 |
| Al (g kg^-1^) | 28 ± 0.6 |
| Ca (g kg^-1^) | 1.9 ± 0.1 |
| Exchangeable Ca (mg kg^-1^) | 501 ± 122 |
| Exchangeable K (mg kg^-1^) | 46.1 ± 12.6 |
| Exchangeable Na (mg kg^-1^) | 25.4 ± 5.1 |
| Available P (mg kg^-1^) | 22.6 ± 6.2 |
| P sorption capacity (mg kg^-1^) | 150 |

The fine and coarse soil fractions obtained from the same Eutric Cambisol soil show very similar profiles. However, the coarse fraction results enriched in Al, K and Ca as shown in Figure S4. The soil pH values are 5.6±0.1 for the coarse fraction and 5.4±0.1 for the fine fraction.


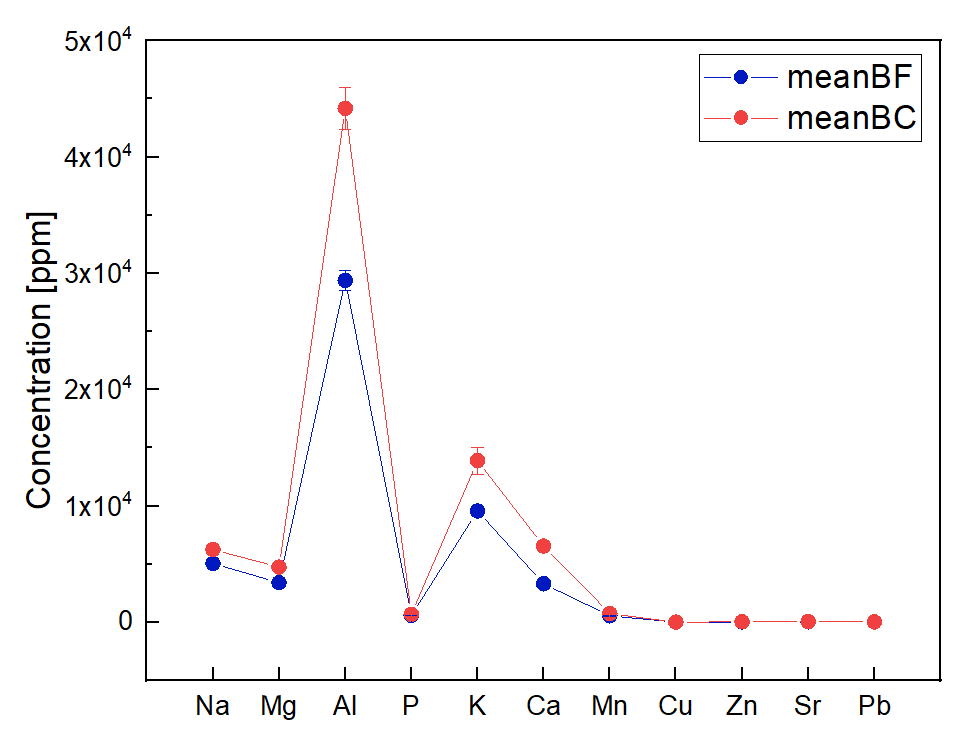


Figure S4 – Concentration of the studied elements in blank soil. Comparison between fine and coarse soil fractions.

4. Calibration


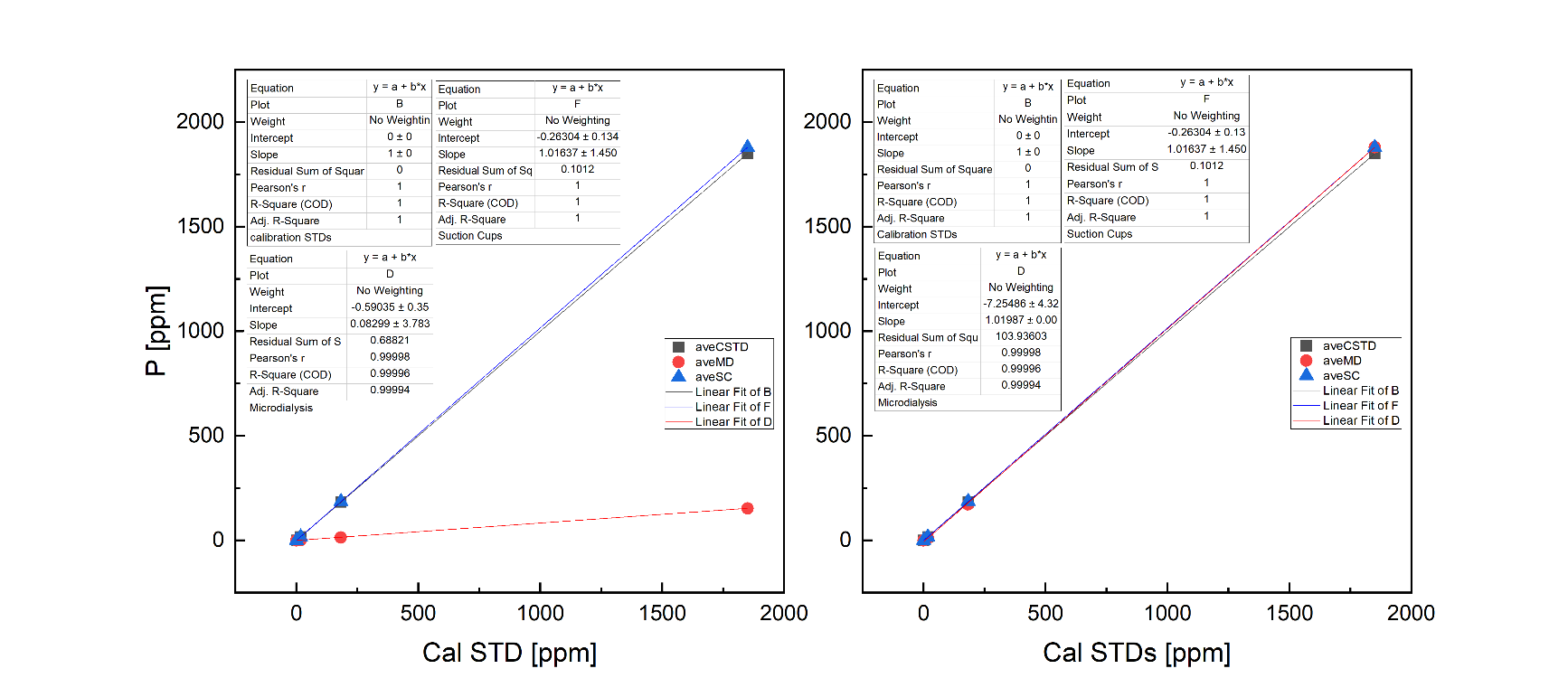


Figure S5 – Calibration curves for microdialysis (red) and suction cups (blue) compared to calibration standards concentrations (grey). Lefthand panel show calibration results; righthand panel show microdialysis calibration after the correction factor was applied

5. Digestion protocol

The samples (100 mg) were first reacted in 3 ml aqua regia overnight. The aqua regia was prepared fresh from sub-boiled nitric and hydrochloric acids purified by sub boiling distillation on Savillex DST-1000 stills from Fisher Primar Plus stock acids. Following drying at 130°C, 2 ml HF (SpA grade, Romil) and 0.75 ml HClO_4_ (Certified for AR grade, Fisher) were added and the mixture left to react overnight at 130°C. The acid mixture was then dried off at 180°C. The samples were then redissolved in 6M HCl on a hotplate at 130°C overnight before being made up to a final mother solution.

6. Diffusion vs gravitation in non-stirred water solutions

We set up two tubes with MilliQ water and a fertilizer pellet positioned on top and on the bottom of the water column, respectively. The observed temporal profiles resulted very different in the two cases and they can be attributed to different physical phenomena. The phosphorus released from the pellet placed on top of the water column goes from 15.3 ppm to 44.6 ppm in the first 80 hours and then equilibrates to a constant value of 38.7 ppm after 150 hours. Since crumbling was observed from the TSP pellet, the terminal velocity for the gravitational settling of a particle falling into a water column was calculated. The timescale of the settling process is compatible with 350nm particles and, therefore, the pellet crumbles gravitational settling is likely to contribute to the observed P concentration trend. When the pellet is on the bottom of the water column, instead, the increase in P concentration detected by the probe is slower and compatible with diffusion timescales.


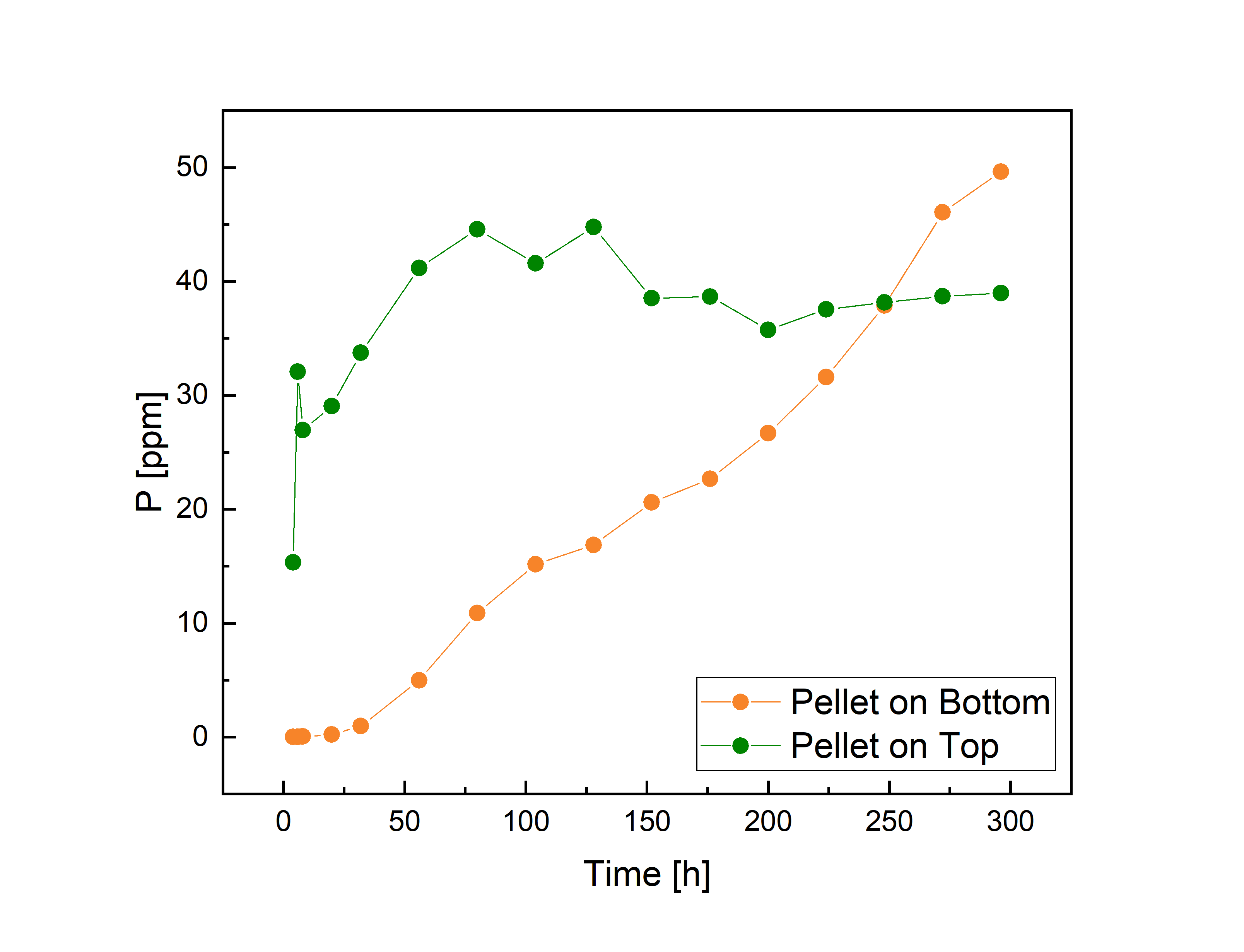


Figure S6 – P concentration trend in non-stirred water columns. The position of the probe above or below the P source determines the different captured phenomena which are diffusion and gravitational settling, respectively.

7. Phosphorus modelling

The following expression for $c_{sol}(z,0)$ was used for parameter estimation:

|  | $c_{sol}\left( z,0 \right)=\left\{ \begin{aligned} c_{pulse}e^{-\left( \frac{z-z_{p}}{a} \right)^{2}}, for z\leq z_{p}, \\ c_{pulse}e^{-\left( \frac{z-z_{p}}{b} \right)^{2}}, for z_{p}<z\leq z_{d,} \\ c_{pulse}e^{-\left( \frac{z_{d}-z_{p}}{b} \right)^{2}}, for z_{d}<z\leq L, \end{aligned} \right.$ | (S1) |
| --- | --- | --- |

where $c_{pulse}$ is the maximum strength of the initial concentration pulse. For our case, the initial position $z_{p}$ of the fertilizer pellet is 17.5 mm from the top of the soil column. Comparing with the soil digestion data, the value of$z_{d}$, which is the depth below which the initial concentration is constant, is chosen to be 27.5 mm. The initial distribution profile of dissolved P is shown in Figure S6 where the estimated value of $c_{pulse}$,$a$ and $b$ is used.

The initial distribution of the concentration of adsorbed P, that is $c_{ad}(z,0)$, is taken to be the pre-fertilization equilibrium value of

|  | $c_{ad}(z,0)=\frac{\beta_{1}}{\beta_{2}}c_{pf}$ | (S2) |
| --- | --- | --- |

where we take the pre-fertilization dissolved P concentration $c_{pf}$ to be $15ppb$ along the entire column.

Figure S6 shows the initial distribution of the dissolved P corresponding to the parameters estimated by modelling. This distribution is based on total P concentrations determined at the end of the experiment by total soil digestion.


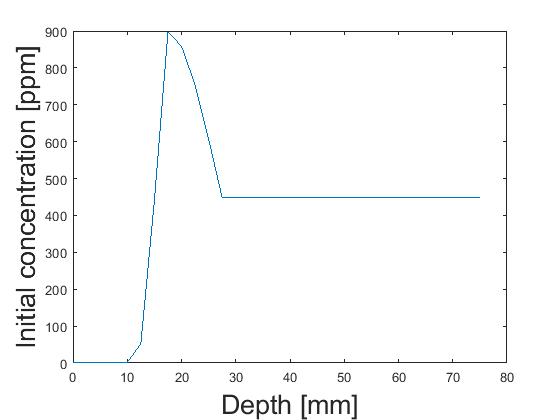


Figure S7 - Initial distribution of dissolved P concentrations along the soil column.
